# Retinoid orphan nuclear receptor alpha (RORα) suppresses the epithelial–mesenchymal transition (EMT) by directly repressing Snail transcription

**DOI:** 10.1016/j.jbc.2022.102059

**Published:** 2022-05-20

**Authors:** Gaofeng Xiong, Ren Xu

**Affiliations:** 1 Markey Cancer Center, University of Kentucky, Lexington, Kentucky, USA; 2 Department of Pharmacology and Nutritional Sciences, University of Kentucky, Lexington, Kentucky, USA

**Keywords:** nuclear receptor, breast cancer, RORE, stemness, Snail, AR, androgen receptor, ChIP, chromatin immunoprecipitation, DMEM, Dulbecco's modified Eagle's medium, EGF, epidermal growth factor, EMT, epithelial–mesenchymal transition, ER, estrogen receptor, HEK 293, human embryonic kidney 293 cell line, HRP, horseradish peroxidase, IgG, immunoglobulin G, RORα, retinoid orphan nuclear receptor alpha, RORE, ROR response element, TGF-β, transforming growth factor-β

## Abstract

Retinoid orphan nuclear receptor alpha (RORα) is a member of the orphan nuclear factor family and regulates gene expression by binding to ROR response elements (ROREs). RORα has been identified as a potential tumor suppressor; however, how downregulation of RORα promotes cancer progression is not fully understood. Here, we showed that protein levels of RORα were downregulated during the Snail-, Twist-, or transforming growth factor-β–induced epithelial–mesenchymal transition (EMT). We found that silencing of RORα induced expression of mesenchymal markers in MCF10A cells, accompanied by enhanced cell invasion, migration, and mammosphere formation. Furthermore, ectopic expression of RORα suppressed transforming growth factor-β–induced EMT processes in MCF10A and HMLE cells. These results indicate that downregulation of RORα is crucial for the induction of EMT in mammary epithelial cells. By analyzing gene expression profiles in control and RORα-expressing cells, we also identified Snail, a key regulator of EMT, as a potential target of RORα. We show that RORα expression significantly inhibits Snail transcription in breast cancer cells. Chromatin immunoprecipitation analysis demonstrated that RORα bound to the ROREs in promoter region of *SNAI1* gene, and using the luciferase reporter assay, we showed that binding to the ROREs was critical for RORα to repress Snail transcription. Finally, rescue experiments substantiated that Snail mediates RORα function in suppressing EMT and mammosphere formation. These results reveal a novel function of RORα in suppressing EMT and identify Snail as a direct target of RORα in mammary epithelial cells.

The epithelial–mesenchymal transition (EMT) is a process characterized by the loss of epithelial characteristics and the acquisition of a mesenchymal phenotype. This process is crucial for normal development such as embryogenesis and organ development, and for pathologic conditions such as wound healing and tumor progression (1, 2, 3). It is well established that EMT dynamics drive cancer progression and metastasis by enhancing cancer cell migration, invasion, and stemness (4, 5). Therefore, inhibition of the EMT is considered a potential strategy for suppressing cancer progression.

Given the important function and dynamic nature of EMT, this cellular event is controlled by a number of EMT inducers, such as transforming growth factor-β (TGF-β), Snail, Slug, ZEBs, and Twists (6). The Snail family are DNA-binding zinc finger proteins and play a fundamental role in EMT by suppressing E-cadherin expression in epithelial cells (7). Three Snail family proteins (Snail, Slug, and Snail3) have been identified in vertebrates (8). The expression of Snail in breast carcinomas is associated with tumor recurrence, metastasis, and poor prognosis (9, 10). To maintain epithelial structure and function, the expression or activity of Snail and other EMT inducers is normally repressed, which suggests the presence of a potential EMT suppressor in normal mammary epithelial cells. Despite recent progress in studying function of EMT in cancer progression, we know little about these suspected EMT suppressors and how these EMT suppressors and inducers regulate the dynamic EMT process in a coordinated fashion.

Nuclear receptors, a family of ligand-dependent transcription factors, regulate gene expression by directly binding to the *cis* response elements in the regulatory regions. Retinoid orphan nuclear receptor alpha (RORα) is considered a member of the orphan nuclear factor family because its ligand has not been well characterized (11, 12). RORα regulates gene transcription by binding to ROR response elements (ROREs) (11, 12). It plays critical roles in many physiological processes, including cell differentiation, metabolism, inflammation, transformation, and circadian rhythm (13, 14, 15, 16, 17). The *RORA* gene maps to 15q22.2, a region that is often deleted in cancer (18). We and others have identified RORα as a potential tumor suppressor in colon cancer, hepatocellular carcinoma, prostate cancer, glioma, and breast cancer (16, 17, 19, 20, 21, 22). We showed that downregulation of RORα is associated with poor clinical outcomes and that reduced RORα expression promotes tumor growth and cancer cell invasion (17). A recent study suggests a potential function of RORα in suppressing EMT phenotypes of glioblastoma cells (23). However, the molecular mechanism by which RORα suppresses EMT remains to be determined.

In the present study, we show that RORα expression is suppressed during the EMT, and that this reduction is sufficient to induce mesenchymal phenotypes in mammary epithelial cells. We also found that RORα inhibited EMT and mammosphere formation by directly repressing Snail transcription. These results identified RORα as a novel EMT suppressor and provided additional insight into roles of RORα in breast cancer progression.

## Results

### RORα expression is downregulated in EMT cells

**Figure 1:**
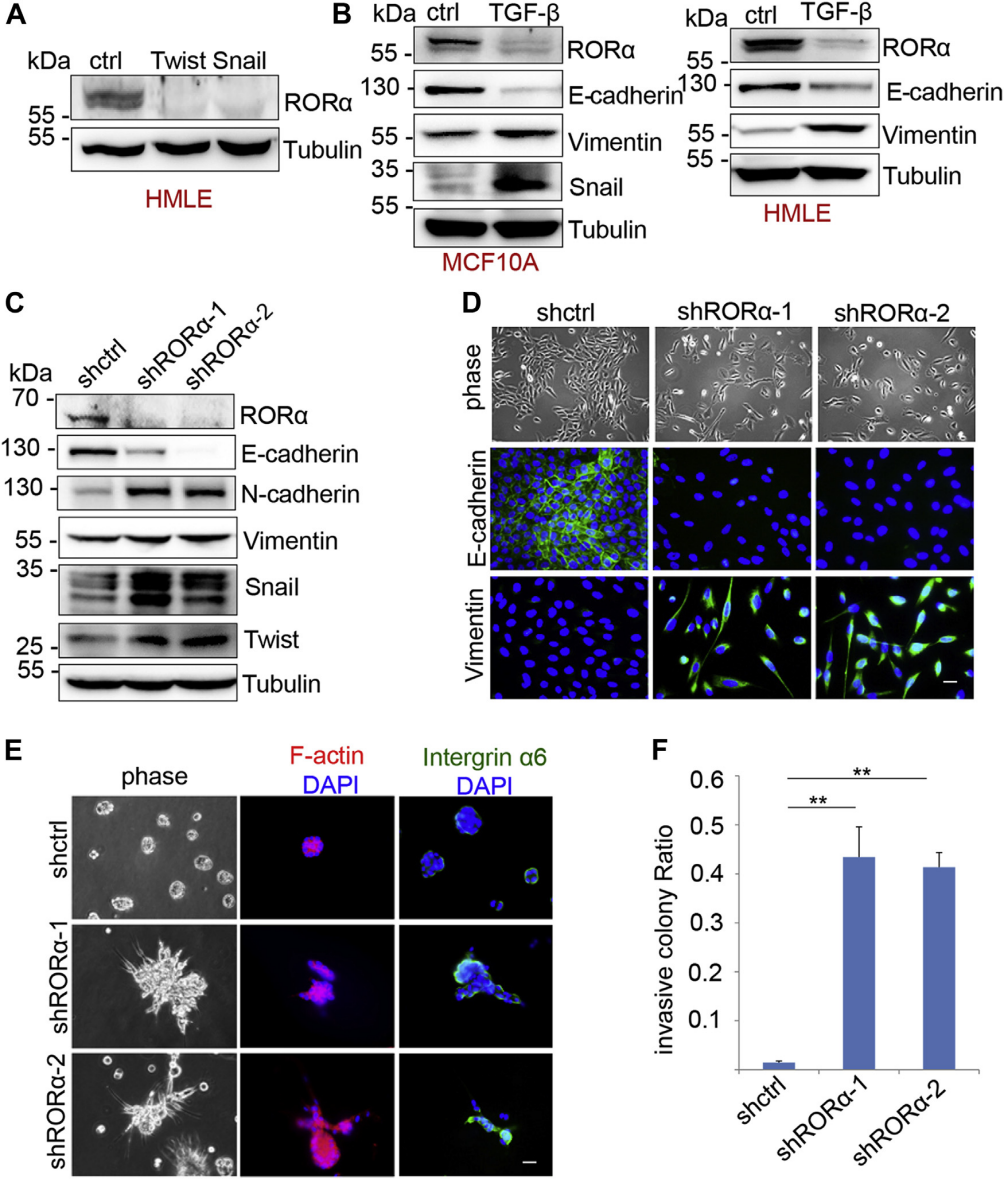
**RORα expression is downregulated in cells that undergo EMT.***A*, protein levels of RORα were examined by immunoblotting in Snail- and Twist-expressing HMLE cells. *B*, protein levels of RORα were examined by immunoblot in MCF10A and HMLE cells treated with TGF-β (10 ng/ml). *C*, EMT marker proteins were examined in control and RORα-silenced MCF10A cells. *D*, phase contrast and immunofluorescence images of control and two knockdown MCF10A cell lines (shRORα-1 and shRORα-2) in 2D culture. Bar represents 10 μm. *E*, phase contrast and immunofluorescent images of control and RORα-silenced MCF10A cells in 3D culture. Bar represents 10 μm. *F*, quantification of invasive colony ratio of control and RORα-silenced MCF10A cells in 3D culture; n = 5. ∗∗*p* < 0.01. EMT, epithelial–mesenchymal transition; RORα, retinoid orphan nuclear receptor alpha; TGF-β, transforming growth factor-β.

Twist and Snail are two transcriptional factors that induce EMT in mammary epithelial cells (8, 24, 25). Stable expression of Snail- or Twist in HMLE cells or MCF10A cells induces the EMT phenotype (25). RORα protein levels were reduced in MCF10A and HMLE cells during the Snail- or Twist-induced EMT (Figs. 1*A* and S1*A*). TGF-β1 treatment induced EMT phenotypes (Fig. S1*B*) and induced EMT marker expression in MCF10A and HMLE cells (Fig. 1*B*). RORα protein levels were also decreased in TGF-β1-treated cells (Fig. 1*B*). Treatment with SB431542, a specific inhibitor of TGF-β type I receptor (26), rescued the RORα expression in TGF-β-treated cells (Fig. S1*C*). These data indicate that reduced RORα protein expression is associated with the EMT process.

### Silenced RORα expression promotes EMT

To determine whether the reduction in RORα expression is functionally significant for the EMT process, we silenced RORα expression with two shRNAs (Fig. 1*C*) and analyzed the EMT phenotypes in those cells. Expression of epithelial cell marker E-cadherin was reduced in RORα-silenced MCF10A cells (Fig. 1*C*). In contrast, expression of mesenchymal cell markers (N-cadherin and vimentin) and the EMT inducers (Snail and Twist) were all upregulated upon RORα knockdown (Fig. 1*D*). Immunofluorescence images further confirmed that silencing RORα in mammary epithelial cells enhanced vimentin expression and induced a spindle-like mesenchymal phenotype (Fig. 1*D*). In 3D Matrigel, RORα-silenced MCF10A cells exhibited an aggressive phenotype with invasive branches, whereas control MCF10A cells formed organized sphere structures (Fig. 1, *E* and *F*). Staining for integrin α6 showed that silenced RORα also disrupted the basal polarity of MCF10A cells in 3D Matrigel (Fig. 1*E*).

**Figure 2:**
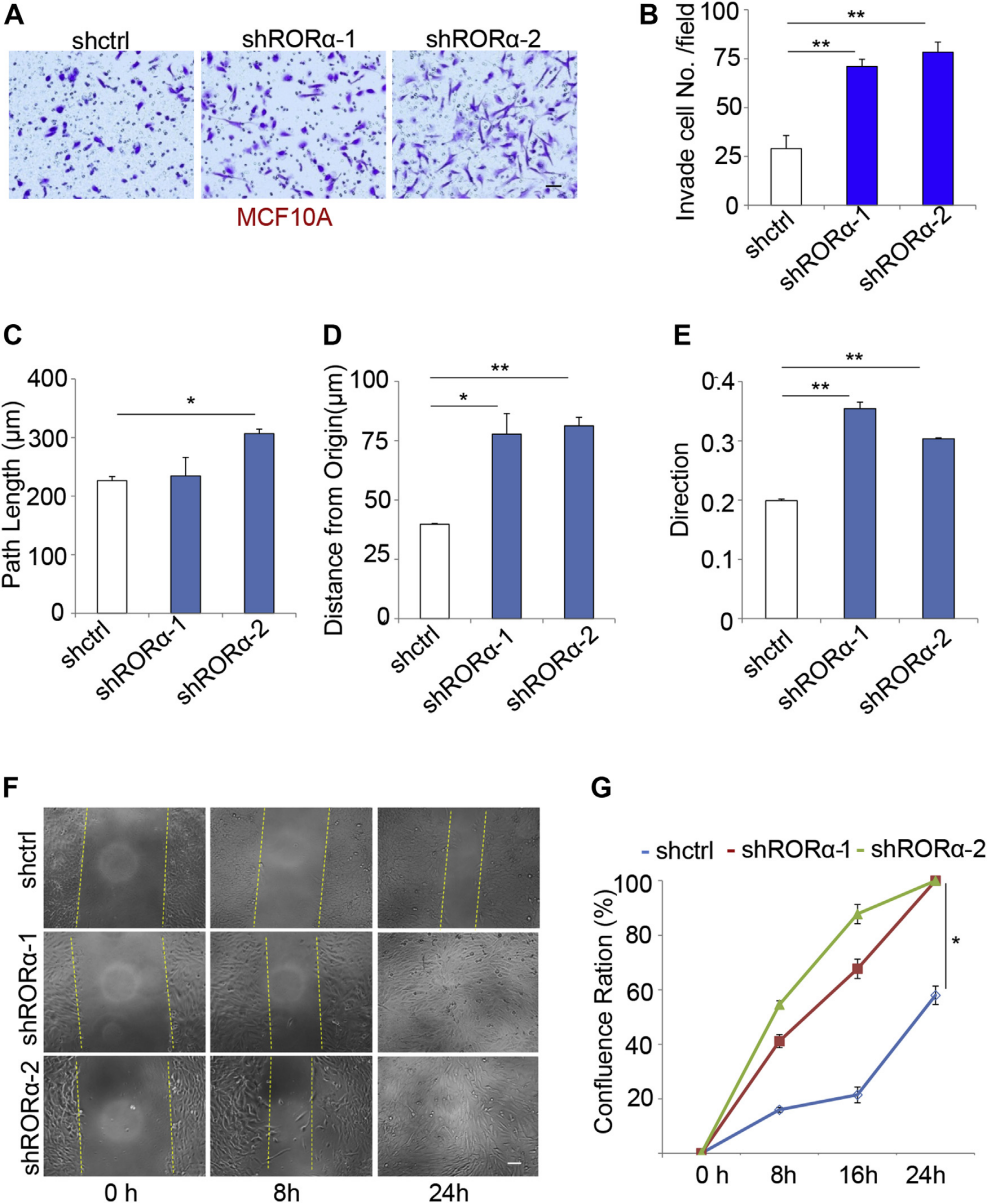
**SilencingRORα expression promotes EMT.***A* and *B*, transwell invasion analysis of control and RORα-silenced MCF10A cells; n = 3. Bar represents 20 μm. ∗∗*p* < 0.01. *C*–*E*, single-cell migration analysis in control and RORα-silenced MCF10A cells. Migration of RORα1 silenced cells was significantly reduced compared with the control cells; n = 100. ∗*p* < 0.01. *F* and *G*, wound healing assay shows the migration of control and RORα-silenced MCF10A cells. Bar represents 20 μm. n = 3. ∗*p* < 0.01. EMT, epithelial–mesenchymal transition; RORα, retinoid orphan nuclear receptor alpha.

It is well established that EMT enhances cell invasion and migration. Consistently, we found that cell invasion was increased in RORα-silenced MCF10A cells (Fig. 2, *A* and *B*). We also tracked single-cell migration using a live cell/incubator imaging system (Fig. S2). Silence of RORα expression in MCF10A cells significantly enhanced cell migration ability (Fig. 2, *C*–*E*). To further confirm our observation, we seeded MCF10A cells in a 96-well culture plate and performed a wound healing experiment. Within 24 h of being scratched, cell confluences were examined by images taken with a Nikon scope (Fig. 2*F*). Silencing RORα in MCF10A cells significantly enhanced cell migration ability and confluence compared with control cells (Fig. 2*G*). These data demonstrate that reduced RORα expression promotes EMT phenotypes in mammary epithelial cells.

### Ectopic RORα expression suppresses the TGF-β-induced EMT

**Figure 3:**
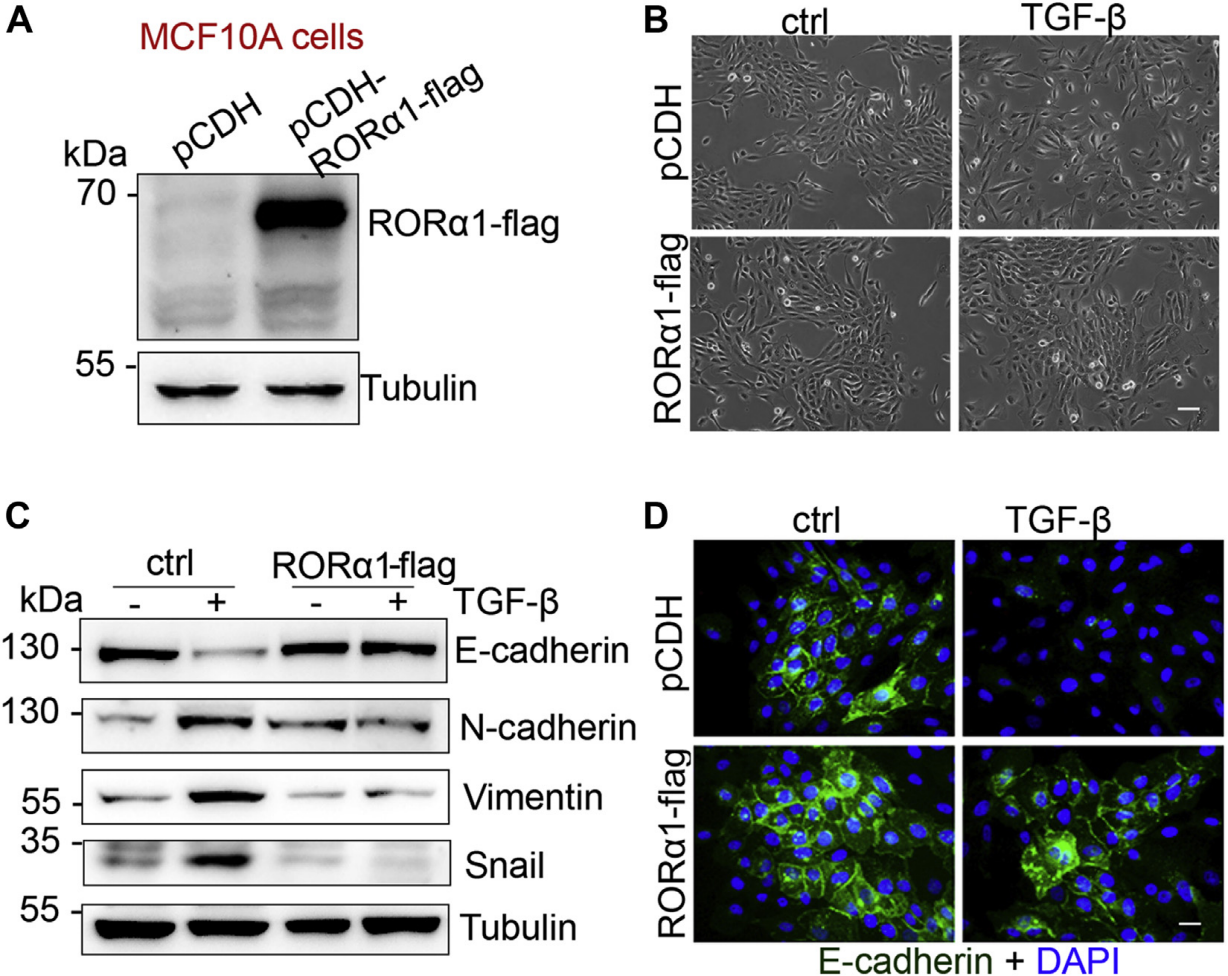
**Ectopic RORα expression suppresses the TGF-β-induced EMT.***A*, RORα expression was verified by Western blot after control, and RORα expression vectors were introduced in MCF10A cells. *B*, phase contrast images of 10 ng/ml TGF-β-treated control and RORα-expressing MCF10A cells in 2D culture. Bar represents 20 μm. *C*, expression of epithelial and mesenchymal marker proteins was examined in 10 ng/ml TGF-β-treated control and RORα-expressing MCF10A cells. *D*, immunofluorescent images showing E-cadherin in the 10 ng/ml TGF-β-treated control and RORα-overexpressing MCF10A cells. Bar represents 10 μm. EMT, epithelial–mesenchymal transition; RORα, retinoid orphan nuclear receptor alpha; TGF-β, transforming growth factor-β.

To examine the role of RORα in suppression of EMT, we introduced exogenous RORα in MCF10A cells (Fig. 3*A*). We treated both vector control MCF10A cells and RORα-expressing MCF10A cells with TGF-β1 (10 ng/ml) for 6 days. Control cells transformed into spindle-like fibroblastic morphology after TGF-β1 treatment, whereas RORα-expressing MCF10A cells maintained an epithelial cell morphology (Fig. 3*B*). We also found that RORα expression suppressed the TGF-β1-induced downregulation of E-cadherin (Fig. 3, *C* and *D*). Expression of mesenchymal markers (N-cadherin, vimentin, and Snail) was induced in control MCF10A cells upon TGF-β1 treatment, whereas their protein levels had no noticeable change in RORα-expressing MCF10A cells (Fig. 3*C*). Similar results were also obtained in HMLE cells (Fig. S3, *A*–*D*). These results suggest that a high RORα protein level in mammary epithelial cells suppresses the TGF-β-induced EMT.

### RORα suppresses stemness in mammary epithelial cell

**Figure 4:**
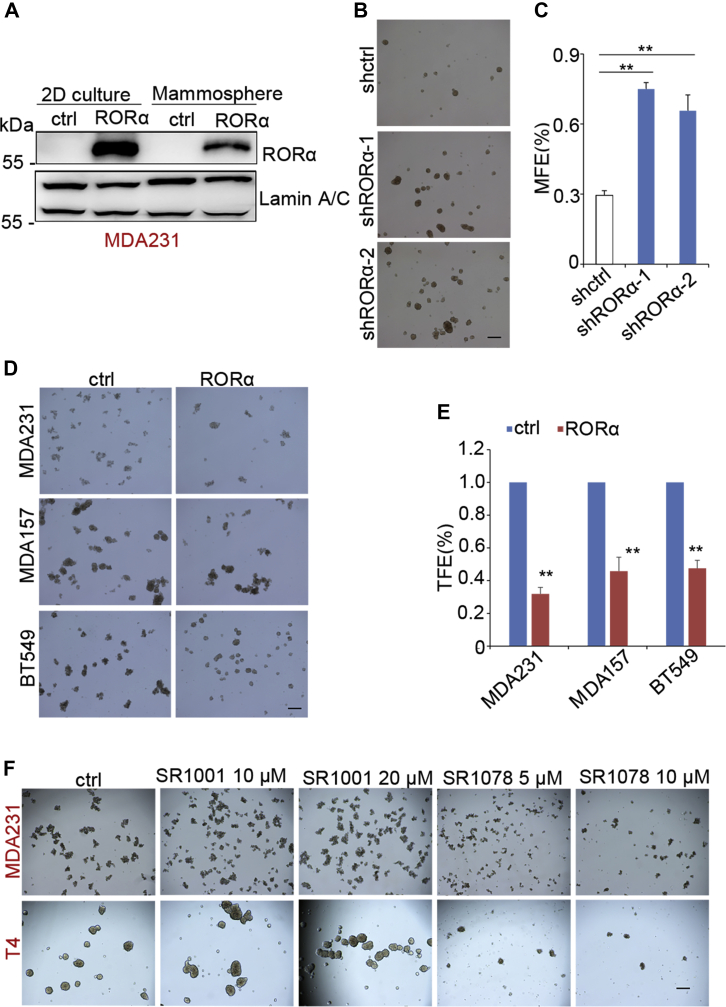
**RORα suppresses mammosphere formation.***A*, RORα expression in 2D cultured cells and mammospheres was examined by Western blot. *B* and *C*, analysis of mammosphere formation in control and RORα-silencing MCF10A cells. Bar represents 100 μm. n = 3. ∗*p* < 0.01. *D* and *E*, tumorsphere assay analyzing the tumorsphere formation in control and RORα-expressing MDA-MB 231 cells, MDA-MB 157 cells, and BT549 cells. Bar represents 100 μm; n = 3. ∗*p* < 0.01. *F*, analysis of tumorsphere-forming abilities of MDA-MB 231 cells or HMT-3522 T4-2 cells (T4) that were pretreated with the RORα antagonist SR1001 (10 μM, 20 μM) or agonist SR1078 (5 μM, 10 μM) in pHEMA-treated (nonattached) plates and incubated in 37 °C, 5% CO_2_, for 5 days. Bar represents 100 μm. pHEMA, poly(2-hydroxyethyl methacrylate); RORα, retinoid orphan nuclear receptor alpha.

It has been shown that EMT is accompanied with enhanced tumor-initiating potential and cancer cell stemness (4, 5). The sphere formation assay has been widely used to enrich cancer stem cells and examine the tumor-initiating capacity of cancer cells (27). RORα protein levels were decreased in tumorspheres compared with levels in cells from 2D cultures (Fig. 4*A*), which suggests a potential function of RORα in regulating cancer cell stemness. Using the sphere formation assay, we showed that silenced RORα significantly increased the efficacy of mammosphere formation in MCF10A cells (Fig. 4, *B* and *C*), whereas ectopic expression of RORα suppressed tumorsphere formation in MDA-MB 231 cells, MDA-MB 157 cells, and BT549 cells (Fig. 4, *D* and *E*). MCF10A-EMT cells (TGF-β induced) exhibited increased mammosphere-forming efficiency, whereas expression of RORα abolished TGF-β1-enhanced mammosphere-forming abilities (Fig. S4, *A* and *B*). Treatment with the RORα antagonist (SR1001) enhanced tumorsphere formation efficiency in MDA-MB 231 and HMT-3522 T4-2 cells, whereas the RORα agonist (SR1078) depressed tumorsphere formation in breast cancer cells (Figs. 4*F* and S4, *C* and *D*). These results suggest that reduction of RORα expression contributes to EMT-associated cancer cell stemness.

### RORα suppresses Snail expression at the transcription level

**Figure 5:**
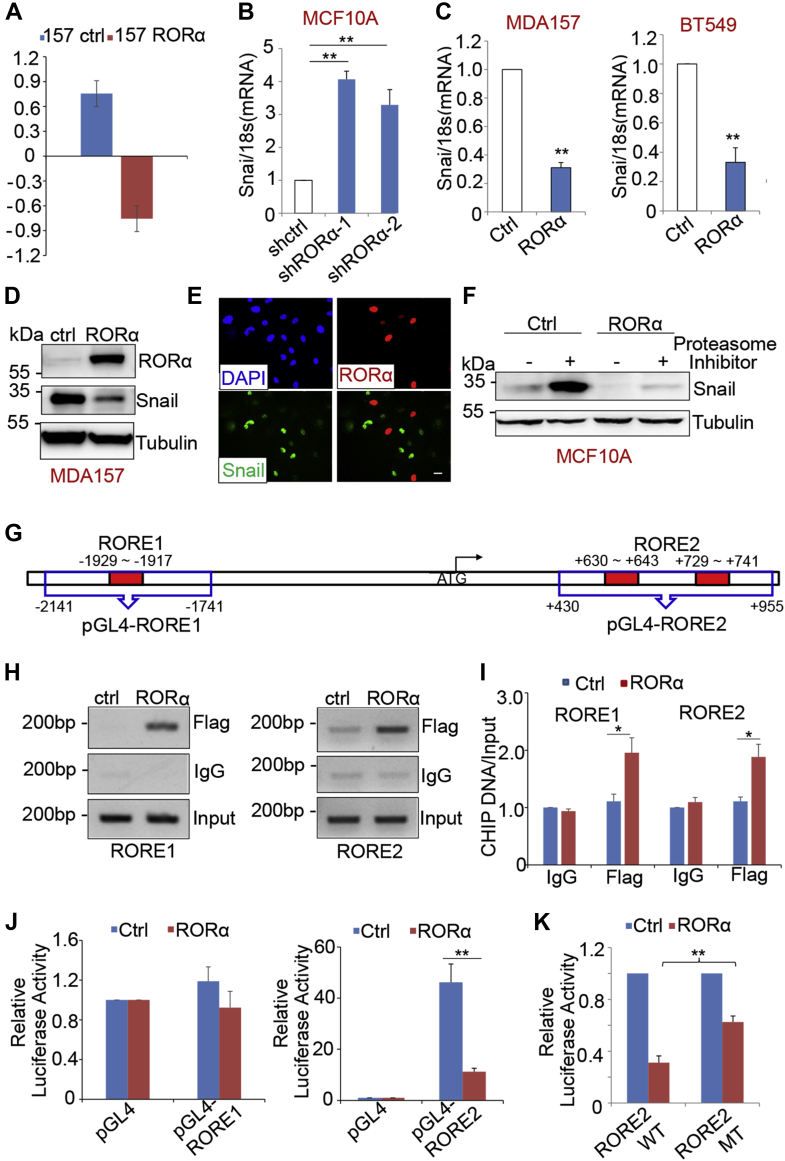
**RORα inhibits Snail expression by directly repressing Snail transcription.***A*, *SNAI1* mRNA levels in control and RORα-expressing MDA-MB 157 cells from the microarray data. *B*, *SNAI1* mRNA levels were quantified by real-time PCR in control and RORα-silenced MCF10A cells; n = 4. ∗∗*p* < 0.01. *C*, *SNAI1* mRNA levels were examined by real-time PCR in control and RORα-expressing MDA-MB 157 and BT549 cells; n = 3. ∗∗*p* < 0.01. *D*, protein expression of Snail was examined by immunoblot of RORα-expressing MDA-MB 157 cells. *E*, confocal images showed Snail and RORα immunofluorescence staining in RORα-expressing MDA-MB 157 cells. Bar represents 10 μm. *F*, Snail protein levels were examined by Western blot in control or RORα-expressing MCF10A cells after treatment with the proteasome inhibitor bortezomib (1 μM) for 24 h. *G*, potential ROREs were identified in *SNAI1* gene. *H*, agarose gel images of ChIP PCR. *I*, ChIP PCR quantification data showed the binding of RORα to ROREs in the *SNAI1* gene in RORα-expressing MDA-MB 231 cells; n = 3. ∗*p* < 0.05. *J*, luciferase reporter data showed that RORα inhibited the transcription driven by RORE2 in HEK 293 cells transfected with renilla luciferase vector, pCDH1-RORA-FLAG and pGL4-RORE2; n = 4. ∗∗*p* < 0.01. *K*, luciferase report data showed that deletion of ROREs in the pGL4-RORE2 vector released the RORα-suppressed transcription in HEK 293 FT cells. The cells were transfected with renilla luciferase vector, pCDH1-RORA-FLAG, and wildtype/mutant pGL4-RORE2 vectors (pGL4-RORE2-WT/pGL4-RORE2-MT); n = 3. ∗∗*p* < 0.01. ChIP, chromatin immunoprecipitation; HEK 293, human embryonic kidney 293 cell line; RORα, retinoid orphan nuclear receptor alpha; RORE, ROR response element.

To determine how RORα suppresses the EMT process, we analyzed gene expression profiles derived from control and RORα-expressing cells (28). Among the downregulated genes, Snail has been identified as a major EMT inducer (6). Therefore, we focused on *SNAI1* as a potential RORα target in EMT regulation (Fig. 5*A*). Real-time PCR analysis confirmed that *SNAI1* mRNA levels were decreased in RORα-expressing cells, whereas silenced RORα increased *SNAI1* transcription (Figs. 5, *B* and *C* and S5*A*). Consistently, treatment with a RORα agonist (SR1078) reduced *SNAI1* mRNA levels in T4 cells, whereas RORα antagonist (SR1001) treatment induced *SNAI1* mRNA expression in a dose-dependent manner in MDA-MB 157 cells (Fig. S5, *B* and *C*). Snail protein levels were also significantly decreased in RORα-expressing mammary epithelial cells (Fig. 5*C*). Confocal images showed that nuclei with RORα staining had a relatively low Snail accumulation in MDA-MB-157 cells (Fig. 5*E*), indicating that Snail is rarely coexpressed with RORα. Treatment with the proteasome inhibitor (Bortezomib) did not rescue Snail expression in RORα-expressing cells (Fig. 5*F*), which suggests that the regulation is not through protein degradation.

By analyzing the DNA sequence of the *SNAI1* gene, we identified three potential ROREs: (i) −1929 to −1917, (ii) + 630 to +643, and (iii) +729 to +741 (Fig. 5*G*). Chromatin immunoprecipitation (ChIP) analysis showed that RORα bound to the ROREs in these regions (Fig. 5, *H* and *I*). To determine whether these ROREs are functionally important for RORα suppression of Snail transcription, we generated luciferase reporter constructs containing these regulatory regions (Fig. 5*G*). The luciferase reporter data showed that RORα suppressed transcription driven by RORE2 (+630 to +643, +729 to +741) in the *SNAI1* gene (Fig. 5*J*). To further verify whether the binding of RORα to this region is functionally important, we deleted the RORE sequences (+630 to +643, +729 to +741) in the RORE2 reporter construct. Deletion of ROREs (+630 to +643, +729 to +741) partially released RORα-dependent transcriptional suppression (Fig. 5*K*). In addition, the luciferase reporter vector pGL4-RORE2 contains a 526 bp DNA region of *SNAI1* gene (from +430 to +955), which encompasses the area of RORE2. It is interesting that deletion of two potential ROREs only partially rescued the luciferase activity, suggesting that unidentified ROREs may exist in this region. Nonetheless, these results indicate that RORα represses Snail expression by directly binding to ROREs in the regulatory regions of *SNAI1*.

### Knockdown Snail expression rescues EMT induced by silenced RORα

**Figure 6:**
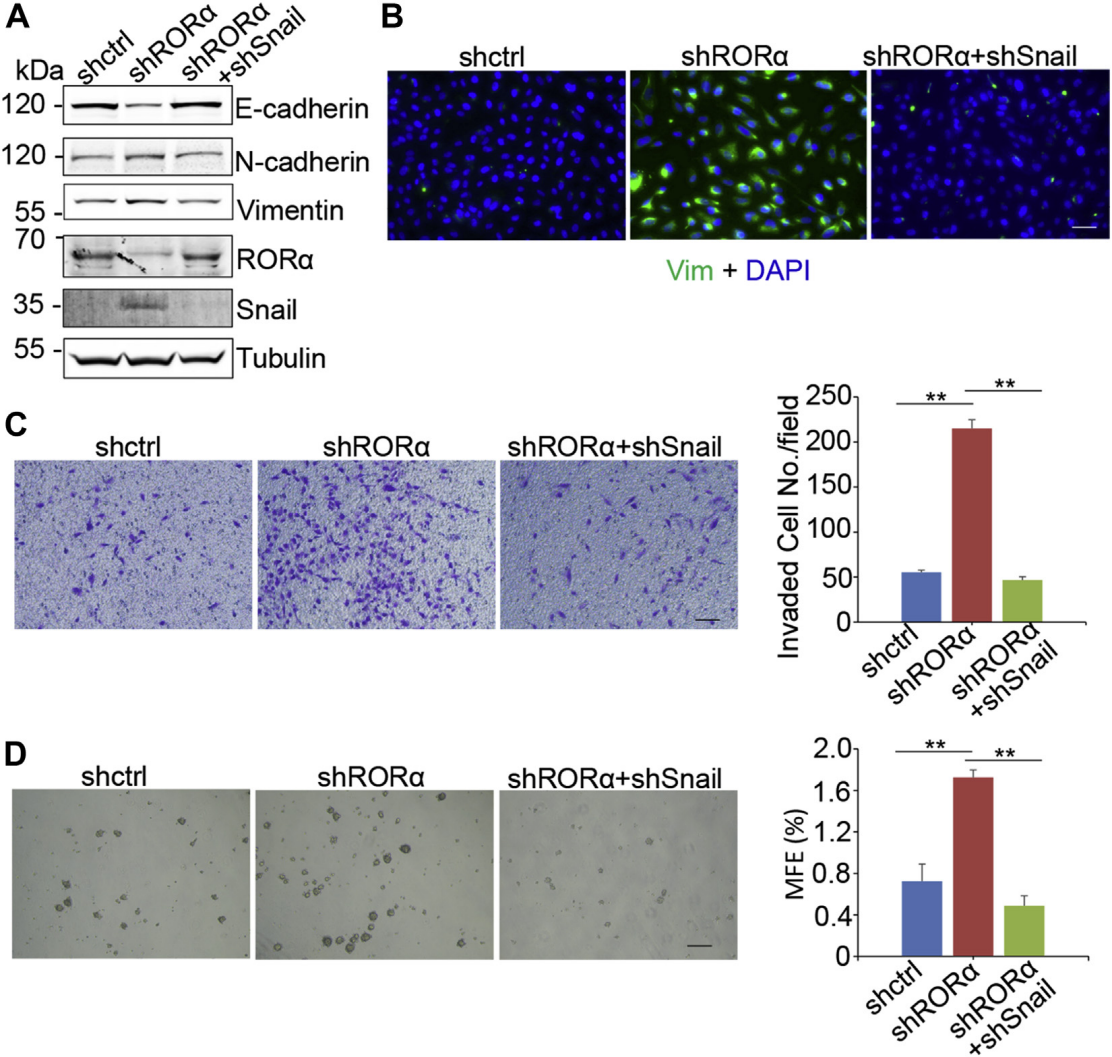
**Knockdown Snail expression rescues EMT induced by silencing of RORα.***A*, EMT marker proteins, RORα and Snail, were examined in control, RORα-silenced, and Snail-/RORα-silenced MCF10A cells. *B*, immunofluorescence images of RORα-silenced and Snail-/RORα-silenced MCF10A cells. Bar represents 50 μm. *C*, transwell invasion analysis of control, RORα-silenced, and Snail-/RORα-silenced MCF10A cells; n = 4. ∗∗*p* < 0.01. Bar represents 100 μm. *D*, analysis of mammosphere formation in control, RORα-silenced, and Snail/RORα both silenced MCF10A cells. n = 4. ∗∗*p* < 0.01. Bar represents 200 μm. EMT, epithelial–mesenchymal transition; RORα, retinoid orphan nuclear receptor alpha.

To investigate whether repression of Snail by RORα is required for the function of RORα in suppressing EMT/stemness, we knocked down Snail expression with shRNA in RORα-silenced MCF10A cells (Fig. 6*A*). Western blot and immunofluorescence results showed that reducing Snail expression in RORα-silenced cells rescued E-cadherin expression and reduced the protein levels of N-cadherin and vimentin (Fig. 6, *A* and *B*). We also evaluated cell invasion and stemness in these cells. Silencing of Snail significantly inhibited cell invasion and repressed stemness in shRORα-expressing MCF10A cells (Fig. 6, *C* and *D*). These results demonstrate that RORα suppresses EMT phenotypes by repressing Snail transcription in mammary epithelial cells.

## Discussion

In the present study, we identified RORα as a potent EMT suppressor in mammary epithelial cells by directly repressing Snail transcription. Similarly, two additional nuclear receptors, the androgen receptor (AR) (29, 30, 31) and the estrogen receptor (ER) (32, 33), have been shown to suppress EMT in prostate and breast cancer cells, respectively. In breast cancer, loss of ER is associated with phosphoinositide 3-kinase signaling activation (33). In prostate cancer, AR is reported to suppress prostate cancer cell EMT by regulation of AKT signaling pathway (30). Another report shows that AR directly represses *SNAI1* gene transcription by binding to AR-responsive elements in the *SNAI1* promoter in prostate cancer (31), which is similar to the mechanism we present in this study for breast cancer.

As an important EMT inducer (6), Snail expression is regulated at multiple levels. Several transcriptional factors have been identified to directly regulate *SNAI1* transcription by binding to its promoter region, including signal transducer and activator of transcription 3 (34), NFκB (35), as well as AR (29, 30, 31) and ER (36, 37). Transcription of Snail is also regulated by epigenetic modifications at the promoter or enhancer regions (H3K27me3, H3K36me2) (38, 39) and by miRNA (40, 41, 42, 43). At the protein level, Snail expression is regulated through eukaryotic translation initiation factor 4E cap-dependent translation (44), Y-box binding protein 1 cap-independent translation (45), and proteasome-dependent protein degradation (46, 47).

We found that RORα directly repressed SNAI1 transcription by binding to RORE in the *SNAI1* gene. The rescue experiments indicate that RORα suppresses EMT in mammary epithelial cells at least partially by repressing Snail expression. Spearman correlation analysis in human breast cancer tissue samples showed that there was no correlation between RORα expression and mRNA levels of Snail in human breast cancer tissue samples (Fig. S6*A*). One potential explanation is that Snail expression in breast cancer tissue is regulated by multiple factors; RORα is only one of these factors. Interestingly, we found that the higher ratio of RORα/Snail mRNA level in breast cancer tissues was associated with longer recurrence-free survival (Fig. S6*B*). These results suggest that the suppression of Snail expression by RORα is negatively associated with breast cancer progression. It has been shown that REV–ERB also binds to RORE (48) and often antagonize the action of RORα in many physiological processes (49). In the future, it will be important to examine whether REV–ERB exhibits opposite function in Snail regulation in the future. We showed that RORα expression was downregulated during the Snail/Twist-induced EMT process. These results suggest that RORα expression is suppressed by Snail directly or indirectly. Therefore, the transcriptional regulation between Snail and RORα may form a negative feedback loop.

NFκB is another EMT inducer identified in mammary epithelial cells (50, 51). It has been shown that RORα inhibits NFκB activation and NFκB-dependent transcription. RORα suppresses the NFκB signaling pathway by induction of nuclear factor of kappa light polypeptide gene enhancer in B-cell inhibitor, alpha (IκBα), a major inhibitory protein of the NFκB functions (52). Another study showed that RORα recruited histone deacetylase 3 to NFκB target genes and attenuated NFκB transcriptional activity in intestinal epithelial cells (53). In human glioma cell lines and glioma stem cells, RORα overexpression inhibits proliferation and tumorigenesis by inhibiting the TNF-α-mediated NFκB signaling pathway (22). Therefore, NFκB may also be a potential RORα target that mediates its function in suppressing EMT.

It is well established that epithelial–mesenchymal plasticity is crucial for cancer cell colonization and metastasis at secondary organs. We recently reported that reduced RORα expression in breast cancer tissue is associated with a high incidence of cancer metastasis, and that RORα expression significantly inhibits spreading of metastatic breast cancer cells to distant organs (28). The findings that RORα suppresses EMT and Snail expression provide additional insights in the function of RORα in breast cancer metastasis.

## Experimental procedures

### Antibodies and reagents

Laminin-rich extracellular matrix was purchased from BD Biosciences (BD Matrigel). RORα complementary DNA was purchased from Open Biosystems. The protein degradation inhibitor, bortezomib, was purchased from Selleck. ShRORα plasmids were purchased from Sigma–Aldrich. TGF-β was purchased from Cayman Chemical. SR1001 and SR1078 were also purchased from Cayman Chemical (catalog nos.: 10922 and 16503). The following antibodies were obtained as indicated: anti-RORα (Santa Cruz; catalog no.: sc-28612), anti-Snail (Cell Signaling; catalog no.:4719S), anti-E-cadherin (BD Biosciences; catalog no.: 610181), anti–N-cadherin (BD Biosciences; catalog no.: 610920), anti-vimentin (Thermo Fisher Scientific; catalog no.: MS-129-P), anti-Twist (Santa Cruz; catalog no.: sc-15393), Alexa Fluor 555 Phalloidin (detecting F-actin; Thermo Fisher Scientific; catalog no.: A34055), anti-Integrin α6 (BD Biosciences; catalog no.: 555734), and antitubulin (MilliporeSigma; catalog no.: 05-661).

### Cell culture and virus preparation

HMEL-Snail, HMLE-Twist, and MCF10A-Snail cells are kind gifts from Dr Binhua Zhou’s laboratory. MCF10A cells were maintained in Dulbecco's modified Eagle's medium (DMEM)/F12 (Sigma) with 5% donor horse serum, 20 ng/ml epidermal growth factor (EGF), 10 μg/ml insulin, 0.5 μg/ml hydrocortisone, 100 ng/ml cholera toxin, and 10 units/ml of penicillin and 0.1 mg/ml of streptomycin (Invitrogen). 3D laminin-rich extracellular matrix cultures were prepared by seeding single cells on top of a thin gel of Matrigel, and subsequently, adding medium containing 5% Matrigel. HMLE cells were maintained in mammary epithelial cell growth medium (Lonza; catalog no.: CC3150). HMT-3522 T4-2 cells (T4 cells) (a kind gift from Dr Mina J Bissell) were maintained on tissue culture plastic as previously described (54). MDA-MB 231 cells were maintained in DMEM/F12 with 10% fetal bovine serum (Sigma–Aldrich). MDA-MB 157 cells and BT549 cells were maintained in DMEM with 10% fetal bovine serum and 10 units/ml of penicillin and 0.1 mg/ml of streptomycin. Human embryonic kidney 293 (HEK 293) FT cells were maintained in DMEM with 10% fetal bovine serum, 0.1 mM nonessential amino acids (Hyclone), 6 mM l-glutamine (Sigma–Aldrich), 1 mM sodium pyruvate (Gibco), 10 units/ml of penicillin and 0.1 mg/ml of streptomycin. All cultured cells were treated with 25 μg/ml plasmocin (InvivoGen) to eliminate and prevent mycoplasma contamination. Cells were cultured at 5% CO_2_ and 95% O_2_ at 37 °C. HEK 293 FT cells were transfected with pCDH1-RORαl-FLAG/shRORα vector (Sigma; TRCN0000022154, TRCN0000022158)/sh*SNAI1* (Addgene; plasmid no.: 115467) plus packaging vectors (pMD2G and psPAX) using Fugene HD Transfection Reagent (Promega). Cancer cells were infected with conditional medium containing lentivirus and selected by puromycin for 1 week 48 h after infection.

### Western blot

Cells were lysed using 2% SDS in PBS buffer containing phosphatase and protease inhibitor cocktails (EMD Millipore; catalog no.: 539131). Protein concentration was measured using Pierce BCA Protein Assay Kit (Thermo Fisher Scientific; catalog no.: 23227). Equal amounts of protein lysates were subjected to SDS gel electrophoresis, immunoblotted with primary antibodies and horseradish peroxidase (HRP)–conjugated secondary antibodies. Depending on the experiment, the secondary antibodies were HRP-conjugated goat anti-rabbit immunoglobulin G (IgG) secondary antibody (Thermo Fisher Scientific; catalog no.: 31460), HRP-conjugated goat antimouse IgG secondary antibody (Thermo Fisher Scientific; catalog no.: 31440), and HRP-conjugated rabbit antigoat IgG secondary antibody (Thermo Fisher Scientific; catalog no.: 31402). The labeled proteins were detected with an enhanced chemiluminescence system (Pierce; catalog no.: 32106).

### Real-time PCR

Total RNA was isolated from cells using Trizol reagent (Invitrogen). The complementary DNA synthesis was performed with SuperScript III First-Strand Synthesis System according to the manufacturer’s instructions. Real-time PCRs were carried out using SYBR Green PCR master mix reagents (Thermo Fisher Scientific) on an ABI 7500 Fast Real-Time PCR System (Applied Biosystems). Thermal cycling was conducted at 95 °C for 30 s, followed by 40 cycles of amplification at 95 °C for 5 s, 55 °C for 30 s, and 72 °C for 15 s. The relative quantification of gene expression for each sample was analyzed by the △△Ct method. The following primers were used to amplify *SNAI1*: 5′-TTTACCTTCCAGCAGCCCTA-3′ and 5′-CCCACTGTCCTCATCTGACA-3′; *18S rRNA*: 5′-ACCTGGTTGATCCTGCCAGT-3′ and 5′-CTGACCGGGTTGGTTT TGAT-3′.

### ChIP assay

ChIP assay was performed based on the Upstate Biotechnology ChIP protocol with a few modifications (55). In general, vector control and RORα-expressing MDA-MB 231 cells were crosslinked with 1% formaldehyde, which was terminated by adding 1.25 M glycine. After formaldehyde crosslinking, nuclei were isolated with a nuclei isolation kit (Sigma) and resuspended in ChIP lysis buffer (1% SDS, 10 mM EDTA, 50 mM Tris–HCl, pH 8.0) containing protease inhibitor cocktail. The absorbance of the lysate at 260 nm was measured and diluted to an absorbance of absorbance of 2 at 260 nm with ChIP lysis buffer. A 20 μl aliquot was removed as input control. Diluted sample was aliquoted into two tubes. To one tube was added 20 μl anti-FLAG M2 beads, and 1 μl IgG antibody was added to the other tube as a negative control. After incubation for 4 h at 4 °C and washed with washing buffer, protein–DNA complexes were eluted from beads and immunoprecipitated according to the Upstate protocol. The isolated DNA was then analyzed by quantitative PCR using the following primers: *SNAI1* promoter: 5′-GGAGAGGAGTCACCTGTTGC-3′ and 5′-TGCTCAGCCTCGTTTAGTGA-3′; 5′-TGGAGACTGGGGA CTTAGGA-3′ and 5′-GGGGCCGATTCTCAATACAT-3′. Agarose gel images were taken by AlphaImager Mini imaging system (ProteinSimple). Data were quantified using AlphaImager software (ProteinSimple) and normalized to input.

### Immunofluorescence

Cells in 3D Matrigel were smeared on slides, dried briefly, and fixed with 4% paraformaldehyde and permeabilized with 0.5% Triton X-100 for 20 min. Cells grown on 2D glass chamber slides (Nunc Lab-Tek II Chamber Slide; Thermo Fisher Scientific) were directly fixed with 4% paraformaldehyde and permeabilized with 0.5% Triton X-100. After being blocked with 10% goat serum at room temperature for 60 min, cells were incubated overnight with primary antibody at 4 °C. After being washed three times, cells were incubated in the dark with fluorescent conjugated secondary antibody for 1 h at room temperature. Stained samples were covered with 4′,6-diamidino-2-phenylindole–containing antifade mounting media (Vector Labs; H-1200-10) and imaged with a Nikon upright epi fluorescence microscope or an Olympus IX81 confocal microscope system.

### Invasion assay and migration assay

Transwell (Corning) membranes were coated with 60 μl 1 mg/ml Matrigel and incubated for 30 min at 37 °C before using. Control or shRORα MCF10A cells (1 × 10^5^ cells in 200 μl medium) were plated on the transwell filters and incubated in 37 °C, 5% CO_2_, for 24 h. Invaded cells on the bottom face of the filter were fixed by methanol and stained with 8% crystal violet. Images were taken with a Nikon microscope, and the number of invaded cells was counted.

For the single-cell migration assay, control or shRORα MCF10A cells (0.04 × 10^6^) were placed on 35 mm dishes (type I collagen precoated) in DMEM/F12 medium containing 2% fetal bovine serum and 4 ng/ml EGF. After 2 h incubation at 37 °C, images were taken using a live cell/incubator imaging system (Nikon Biostation IMQ) every 10 min for 8 h.

### Wound healing

Control or shRORα MCF10A cells were seeded into 96-well tissue culture plate. After 24 h of growth, when cells reached 80 to 90% confluency, crosses were gently scratched in the monolayer with a new 100 μl pipette tip. Cells were cultured for another 24 h. Photos were taken with a Nikon microscope at different time points over the next 24 h period.

### Luciferase reporter assays

A DNA fragment (−2141 ∼ −1741) containing RORE region 1 (−1929 to −1917, RORE1) was amplified from human genomic DNA using primers 5′-GCGGGTACCCCCTGTGGGGATCTAATGTG-3′ and 5′-GCGAAGCTTGCTCTTCCTCCTCTCCCCTA-3′ and then cloned into luciferase report vector pGL4. Another DNA fragment (+430 ∼ +955) containing RORE region 2 (+630 to +643, +729 to +741) was amplified from human genomic DNA using primers 5′-GCGGGTACCGAAGGAGAGGAGGCCTGTGT-3′, 5′-GCGAAGCTTGGACAGAGTCCCAGATGAGC-3′ and cloned into luciferase report vector pGL4. A deletion (RORE region from +630 to +643, from +729 to +741) mutant was constructed by primers 5′-AGATCAGGAACAACTGGGGGTCC TACGTGT-3′, 5′-CCCCAGTTGTTCCTGATCTCCCTCTCCTA-3′, 5′-ATAATTTTTTGTATTGAGAATCGGCCCCAC-3′ and 5′-TTCTCAATACAAAAAATTATCCACAGGACAG-3′. HEK 293 cells were transient transfected with pCDH1-RORA-FLAG, pGL4-RORE1, pGL4-RORE2, or pGL4-RORE2 MT (mutant type) and renilla luciferase vector. Cell lysates were collected for the luciferase assay 48 h after transfection.

### Sphere formation assay

Nonadherent 24-well tissue culture plates were obtained by pretreating with poly(2-hydroxyethyl methacrylate) (12 mg/ml in 95% ethanol) at 53 °C for overnight. Cells grown on 2D plastic culture dish were trypsinized according to standard protocol and filtered by Cell Strainer (40 μm; Corning) to ensure a single-cell suspension. Viable cell numbers were calculated after trypan blue staining using a hemocytometer. Stem cell culture media: DMEM/F12 medium supplemented with B27 (1:50), EGF (20 ng/ml), basic fibroblast growth factor (20 ng/ml), insulin (5 μg/ml), hydrocortisone (0.5 μg/ml), and gentamycin (100 μg/ml) were added into each well of a nonadherent 24-well culture plate. Single-cell suspensions were plated at an appropriate density in triplicate. After 5 days of incubation at 37 °C and 5% CO_2_ without moving or disturbing the plates, the number of mammospheres/tumorspheres greater than 50 μm diameter was counted using a microscope (at ×40 magnification). Mammosphere/tumorsphere-forming efficiency (%) was calculated as follows: number of spheres per well/number of cells seeded per well ×100.

### Statistical analysis

Experiments were repeated at least three times. Results were reported as mean ± standard error of the mean, and the significance of difference was assessed by independent Student’s *t* test. *p* < 0.05 represented statistical significance, and *p* < 0.01 represented sufficient statistical significance.

## Data availability

All data are contained within the article.

## Supporting information

This article contains supporting information.

## Conflict of interest

The authors declare that they have no conflicts of interest with the contents of this article.
