# Supplementary material for: Retinoid orphan nuclear receptor alpha (RORα) suppresses the epithelial–mesenchymal transition (EMT) by directly repressing Snail transcription
Source: J Biol Chem. 2022 May 20;298(7):102059. doi: 10.1016/j.jbc.2022.102059 (PMC9218514; doi:10.1016/j.jbc.2022.102059)
Supplement: Supplemental Figures S1–S6 [file mmc1.docx]

**Supporting information**


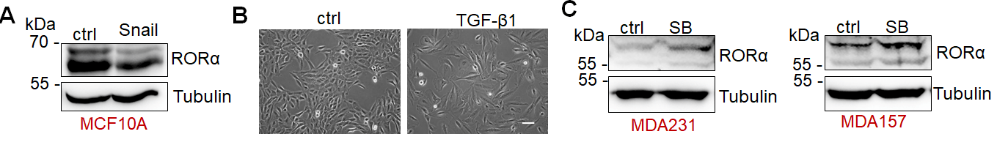


Figure. S1: A) Protein levels of RORα were examined by immunoblotting in Snail induced MCF10A-EMT cells\. B) Phase images of MCF10A cells treated with/without TGF-β1 (10 ng/ml) for 10 days. Bar, 20µm. C) RORα protein level was increased in MDA-MB 231 cells and MDA-MB 157 cells treated with TGF-β1 inhibitor SB431542 (10 μg/ml) for 4 days. SB: SB431542.


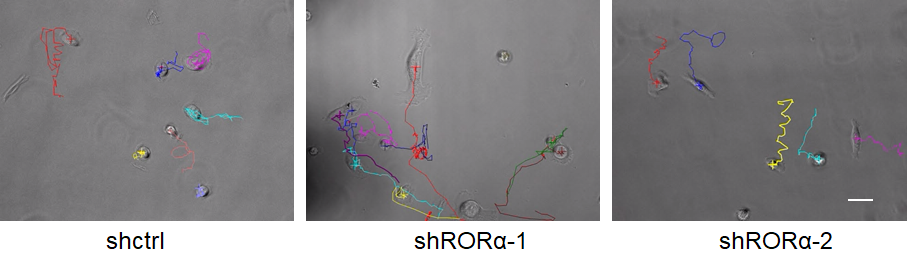


Figure. S2: The paths of tracked single cell migration in control and shRORα MCF10A cells. Bar, 20µm.

.


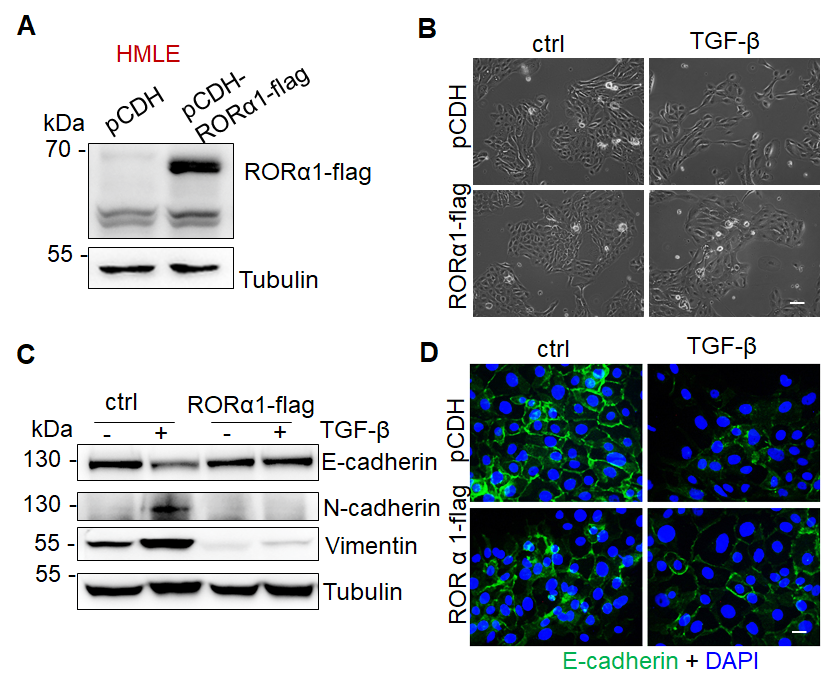


Figure. S3: A) RORα expression in infected HMLE cells was verified by Western blot. B) Phase images of TGF-β treated control and RORα-expression HMLE cells at 2D culture. Bar, 20µm. C) EMT marker proteins were examined in TGF-β treated control and RORα-expression HMLE cells. D) Immunofluorescence images of TGF-β treated control and RORα overexpression HMLE cells. Bar, 10µm.


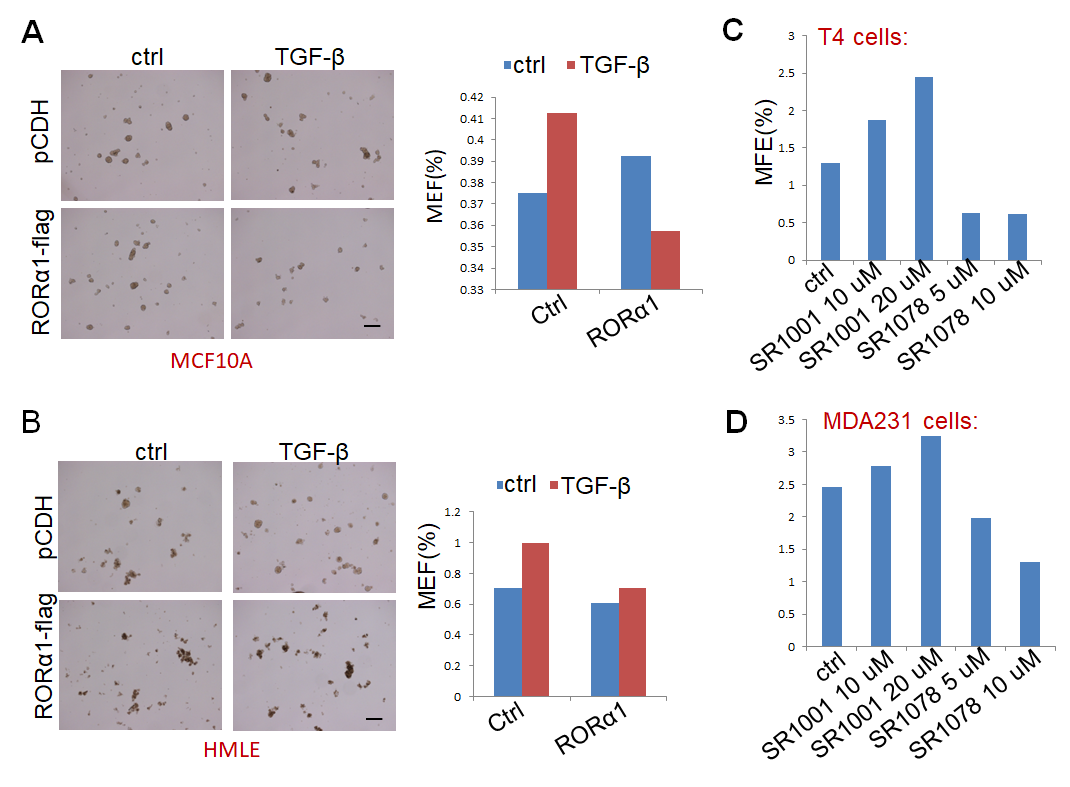


Figure. S4: A) Mammosphere assay analyzing the mammoshpere forming abilities of control and RORα-expression MCF10A cells treated with/without TGF-β1. Bar, 100 µm. B) Mammosphere assay analyzing the mammoshpere forming abilities of control and RORα-expression HMLE cells treated with/without TGF-β1. Bar, 100 µm. C) Quantification tammoshpere forming efficiency of antagonist SR1001 (10 μM, 20 μM) and agonist SR1078 (5 μM, 10 μM) pre-treated T4 cells. D) Quantification tammoshpere forming efficiency of antagonist SR1001 (10 μM, 20 μM) and agonist SR1078 (5 μM, 10 μM) pre-treated MDA-MB 231 cells.


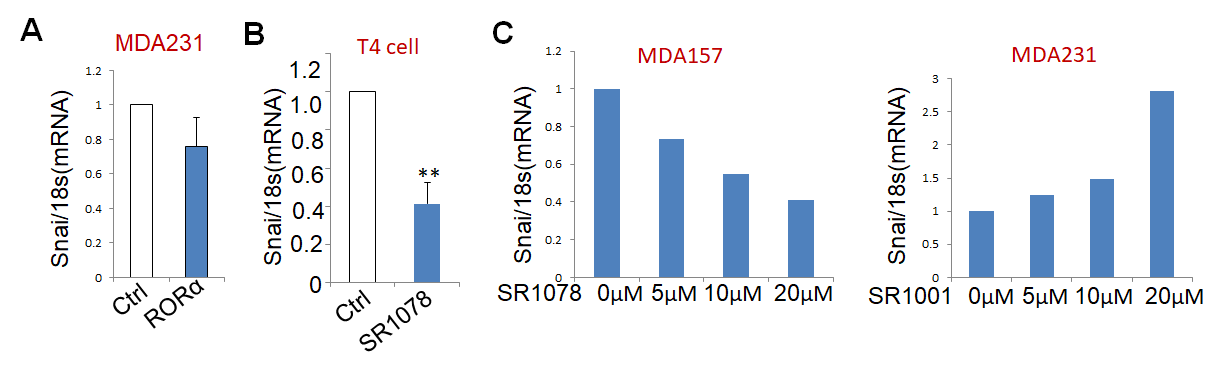


Figure. S5: A) Snail mRNA levels were examined by Q-PCR in control and RORα-expression MDA-MB 231 cells. B) *SNAI1* mRNA levels were examined by Q-PCR in control and and RORα agonist SR1078 treated T4 cells. n=5, **, P < 0.01. C) Snail mRNA levels were examined by Q-PCR in RORα agonist SR1078 (5 μM, 10 μM, 20 μM) treated MDA-MB 157 cells, and RORα antagonist SR1001 (5 μM, 10 μM, 20 μM) treated MDA-MB 231 cells.


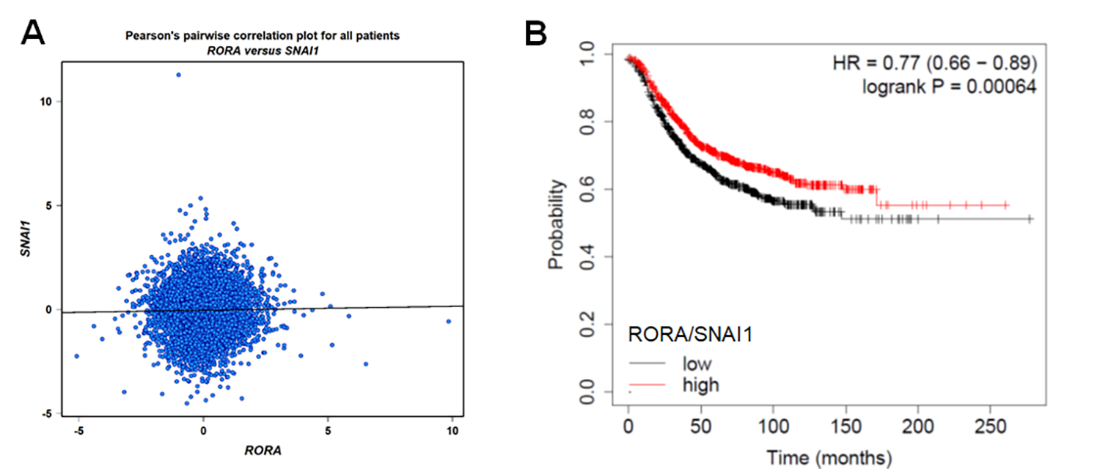


Figure. S6: A) The association between RORα expression and mRNA levels of Snail in human breast cancer tissue samples. n=10059. B) The association of RORα/Snail ratio level with recurrence-free survival was assessed by analyzing the mRNA levels in breast cancer patient tissues, n = 2032. Kaplan–Meier survival analysis.
